# A Dithiin‐Linked Covalent Organic Polymer for Ultrahigh Capacity Half‐Cell and Symmetric Full‐Cell Sodium‐Ion Batteries

**DOI:** 10.1002/advs.202304497

**Published:** 2023-09-25

**Authors:** Shen Xu, Chenchen Wang, Tianyi Song, Huiying Yao, Jie Yang, Xin Wang, Jia Zhu, Chun‐Sing Lee, Qichun Zhang

**Affiliations:** ^1^ Department of Materials Science and Engineering City University of Hong Kong Hong Kong SAR 999077 P. R. China; ^2^ Department of Chemistry City University of Hong Kong Hong Kong SAR 999077 P. R. China; ^3^ School of Chemical Engineering Anhui University of Science and Technology Huainan 232001 P. R. China; ^4^ National Center for Nanoscience Technology (NCNST) No.11 ZhongGuanCun BeiYiTiao Beijing 100190 P. R. China; ^5^ Center of Super‐Diamond and Advanced Films (COSDAF) City University of Hong Kong Hong Kong SAR 999077 P. R. China

**Keywords:** covalent organic polymers, dithiin linkage, Na‐ion batteries, Na‐ion storage mechanism, organic electrodes, symmetric full‐cells

## Abstract

Sodium ion‐batteries (SIBs) are considered as a class of promising alternatives to lithium‐ion batteries (LIBs) to overcome their drawbacks of limited sources and safety problems. However, the lack of high‐performance electrode materials hinders the wide‐range commercialization of SIBs. Comparing to inorganic counterparts, organic electrode materials, which are benefitted from flexibly designable structures, low cost, environmental friendliness, and high theoretical gravimetric capacities, should be a prior choice. Here, a covalent organic polymer (COP) based material (denoted as CityU‐9) is designed and synthesized by integrating multiple redox motifs (benzoquinone and thioether), improved conductivity (sulfur induction), and intrinsic insolubility (rigid skeleton). The half‐cell SIBs exhibit ultrahigh specific capacity of 1009 mAh g^−1^ and nearly no capacity drop after 650 cycles. The first all‐COP symmetric full‐cell shows high specific capacity of 90 mAh g^−1^ and excellent rate capability. This work can extend the selection of redox‐active moieties and provide a rational design strategy of high‐performance novel organic electrode materials.

## Graphical abstract

A covalent organic polymer (COP, CityU‐9) is developed as the electrode material of sodium‐ion batteries (SIBs). This novel electrode material can not only realize specific capacity up to 1009 mAh g^−1^ in half‐cells, but also obtain high capacity of 90 mAh g^−1^ in the first all‐COP symmetric full‐cell SIB, accompanying with excellent rate performance and stability.

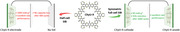

## 1 Introduction

The increasing demand of modern society on clean energy consumption and storage has triggered the explosive development of electrochemical energy storage (EES) devices.^[^ ^1^, ^2^, ^3^, ^4^, ^5^ ^]^ Among them, rechargeable lithium ion batteries (LIBs) have arisen tremendous attentions due to their ultrahigh energy density, and commercialized widely for decades.^[^ ^6^, ^7^, ^8^, ^9^ ^]^ However, the limited lithium sources and safety problems push researchers to seek the alternatives such as sodium ion batteries (SIBs).^[^ ^10^, ^11^, ^12^ ^]^ Developing high‐performance electrode materials is one of the main challenges for the commercialization of SIBs.^[^ ^13^ ^]^ Comparing with inorganic counterparts, organic electrode materials with high theoretical gravimetric capacities are renewable and designable for specific function, which have been considered as one of prior choices.^[^ ^14^, ^15^ ^]^

It is well known that some organic small molecules with redox active groups have been demonstrated to show excellent battery performance when utilized as the electrode materials for SIBs.^[^ ^16^, ^17^, ^18^ ^]^ By carefully optimizing the electrolyte and the morphology of the active materials, Bao et al. investigated the redox mechanism of a previously reported sodium‐organic coordination compound (Na_2_C_6_O_6_) and achieved very high specific capacity of 498 mAh g^−1^.^[^ ^16^ ^]^ A high capacity of nearly 400 mAh g^−1^ could still be retained from the second cycle. Huang and co‐workers utilized calix[4]quinone (C4Q) and pillar[5]quinone (P5Q) as the cathode materials and combined them with CMK‐3 (ordered mesoporous carbon), which is an encapsulating material.^[^ ^17^, ^18^ ^]^ High specific capacities of 422 and 418 mAh g^−1^ were obtained respectively for the initial cycle. Insufficiently, both of them exhibit obvious drops on the capacities from the second cycle. Thus, the organic small molecules still suffer from their solubility in electrolytes, which is disadvantage for long‐term stability of the batteries.^[^ ^14^, ^19^ ^]^

Covalent organic polymers (COPs) such as covalent organic frameworks (COFs) are considered as a promising class of organic electrode materials owing to their rigid structure, easiness of modification, and insolubility.^[^ ^20^, ^21^, ^22^ ^]^ Moreover, the porous backbones endow their sufficient specific surface area and facilitate the efficient ion insertion/extraction during the charging/discharging processes in batteries.^[^ ^23^, ^24^, ^25^ ^]^ For instance, Chen et al. achieved a very high specific capacity of 452 mA h g^−1^ when utilizing TQBQ‐COF with dual active redox sites (C=O and C=N) as the cathode material.^[^ ^22^ ^]^ Notably, a high reversible capacity of ≈400 mAh g^−1^ was maintained for 10 cycles, which is much improved comparing with organic small molecule‐based cathodes. Therefore, employing COPs as the electrode materials for SIBs is promising. However, the researches focusing on COP‐based electrode materials for SIBs are limited.^[^ ^26^, ^27^ ^]^ Much more efforts are still needed to simultaneously realize high capacity and excellent cyclic stability.

In this research, a newly designed thioether‐containing COP, named as CityU‐9, was synthesized and utilized as the electrode active material for high‐performance SIBs. Particularly, the dithiin linkages not only serve as the redox active sites but also favor the improvement of the conductivity. Besides, the well‐known and the most efficient redox units of benzoquinone moieties are also introduced to provide sufficient interacting sites with sodium ions during the insertion and extraction process. With the multiple redox sites, CityU‐9 can act as either the cathode or anode material for half‐cell batteries, and more excitingly as both cathode and anode materials for symmetric full‐cells. The half‐cell SIB shows ultrahigh specific capacity of 1009 mAh g^−1^ at the discharging rate of 50 mA g^−1^. Notably, this battery exhibits no capacity drop after 650 cycles at the high discharging rate of 200 mA g^−1^ with the maximum capacity of 588 mAh g^−1^. Furthermore, the CityU‐9 is utilized as both cathode and anode materials for the first all‐COP symmetric full‐cell SIB with a reversible specific capacity of 90 mAh g^−1^. This full‐cell battery exhibits good cycling stability with a specific capacity retention of 41 mAh g^−1^ over 1000 cycles. In addition to providing high performance electrode material for EES, the novel design concept proposed by this work would expand the vision of design and construction of new electrode materials.

## 2 Results and Discussion

### 2.1 Material Design and Characterization

**Figure 1:**
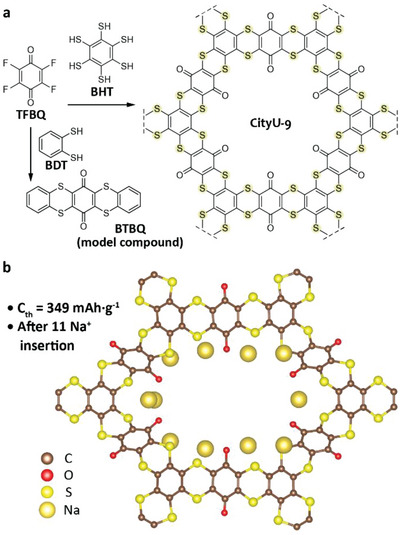
a) Schematic structure and synthetic route of CityU‐9 and model compound BTBQ. b) Calculated Na^+^ insertion positions and theoretical capacity of CityU‐9.

**Figure 2:**
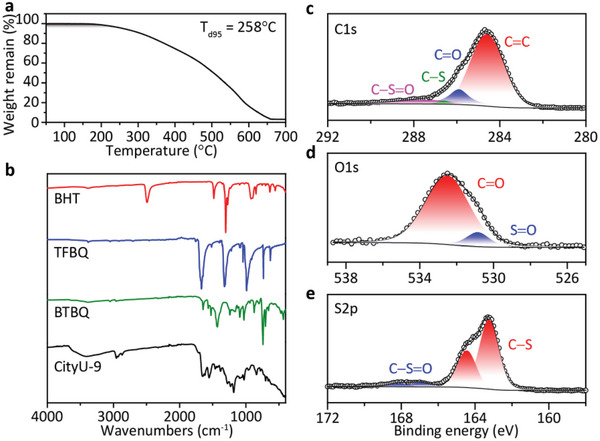
a) Thermogravimetric analysis of CityU‐9. b) FT‐IR spectra of BHT, TFBQ, BTBQ, and CityU‐9. XPS spectra of CityU‐9 including c) C1s, d) O1s, and (e) S2p.

Reducing the ratio of inactive groups (dead mass) in the electrode material is the dominant rule to maximize their theoretical specific capacity.^[^ ^22^ ^]^ Carbonyl‐containing groups (especially benzoquinones) have been widely considered as the most efficient active motifs to capture alkali metal ions for high performance EES devices.^[^ ^14^, ^28^ ^]^ Recently, sulfur‐containing 2D materials have been applied in high performance LIBs,^[^ ^29^, ^30^ ^]^ owing to the proven redox active property of thioether groups and capability of sulfur atoms in improving conductivity.^[^ ^31^, ^32^ ^]^ Based on these considerations, herein, we used sulfur atoms as the bridge to link benzoquinone moieties for the construction of dithiin‐linked COP (namely CityU‐9, **Figure** **1**). This material can be facilely synthesized through the cross‐coupling reaction between 1,2,3,4,5,6‐benzenehexathiol (BHT) and 2,3,5,6‐tetrafluoro‐1,4‐benzoquinone (TFBQ) in the presence of base at 120 °C for 72 h. Further purification via washing with common solvents could afford the desired COP in high yield. The model compound namely BTBQ was synthesized via a similar procedure using benzenedithiol (BDT) and TFBQ as the precursors. The as‐prepared CityU‐9 exhibits excellent thermal stability. The temperature for 95% weight retain (*T*_d95_) is 258 °C, which can ensure good battery thermal stability (**Figure** **2**a).

The structure of CityU‐9 was determined through several characterization tools. As shown in the Fourier transform infrared (FT‐IR) spectra (Figure 2b), the signal of ─S─H bonds (≈2500 cm^−1^) of BHT disappears after the coupling reaction, indicating the full conversion of the precursors.^[^ ^33^ ^]^ Moreover, the newly emerged peaks at 703 and 664 cm^−1^ indicate the formation of the ─C─S bonds, while the peaks at 1233 and 1028 cm^−1^ demonstrate the appearance of = C─S─C bonds. These results illustrate the formation of dithiin linkage, which are in accordance with the peaks of the BTBQ at 705, 658, 1243, and 1030 cm^−1^, respectively. The X‐ray photoelectron spectra (XPS) of CityU‐9 were also measured to determine the material composition. According to the C1s spectra, four peaks can be identified (Figure 2c). The main peak with the binding energy of 284.6 eV comes from the benzene rings and C=C bonds of the benzoquinone moieties. Besides, the small peaks at 287.4 and 285.9 eV can be easily assigned to the C=O bond of benzoquinone groups and the C─S bond, respectively, while the shadow peak at 286.4 eV is contributed from the sulfoxide moiety, which is resulted from the slight oxidation of dithiin linkages.^[^ ^34^ ^]^ To confirm this, the O1s and S2p spectra of CityU‐9 were further investigated. As shown in Figure 2d, the main peak located at 532.5 eV originates from benzoquinone groups, while the shoulder peak at 530.9 eV is consistent with the signal of sulfoxide groups. More convincingly, two couples of 2p peaks can be found in the S2p spectra (Figure 2e). The domain peaks at 163.3 eV refer to the dithiin linkages (2p 3/2), and the shadow peaks at 167.0 eV corresponds to sulfoxide groups (2p 3/2).^[^ ^35^, ^36^ ^]^ We further tested the solid‐state ^13^C nuclear magnetic resonance (NMR) spectrum to prove the structure of CityU‐9. Five peaks can be recognized and assigned to the carbon atoms of CityU‐9 backbone including the carbon connected to sulfoxide group (Figure S1, Supporting Information). All these factors clearly confirm the structure of CityU‐9 but with slight oxidation.

### 2.2 Theoretical Calculations

**Figure 3:**
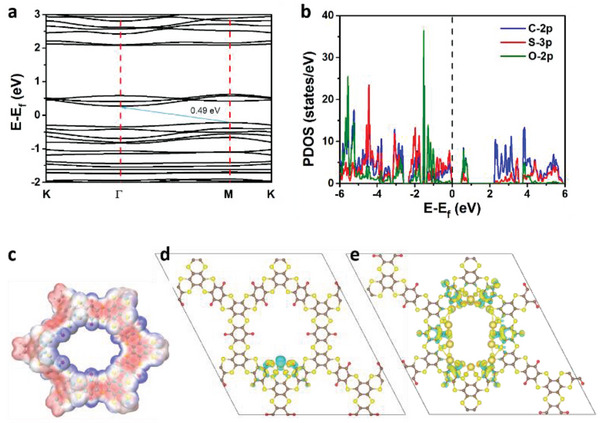
a) Calculated electronic band structure, b) partial density of states (PDOS) of C2p, S3p, and O2p, as well as c) electrostatic potential (ESP, blue color represents the minima) of CityU‐9. Differential charge density of CityU‐9 after d) addition of the first Na ion (isosurface value is 0.002) and e) addition of 11 Na ions (isosurface value is 0.004). Cyan part represents the reduction of charge density, while yellow represents the increase.

The abundant redox active sites of CityU‐9 are in favor of its application as electrodes in metal‐ion batteries. The capability of CityU‐9 as an electrode material in SIB was first evaluated by quantum calculation. As illustrated in **Figure** **3**a, the electronic band structure of CityU‐9 illustrates indirect gap nature with the energy gap of 0.48 eV. This energy gap is in accordance with the experimental result obtained from diffuse reflectance spectrum (Figure S2, Supporting Information). Afterward, the partial density of states (PDOS) was calculated to evaluate the contribution of the individual elements (Figure 3b). The carbon and sulfur atoms participate in the construction of valence band (VB), whereas oxygen and carbon contribute to the conduction band (CB) most. The DFT calculation on the frontier molecular orbitals (FMOs) of the mono ring in Figure S3 (Supporting Information) reveals that the highest occupied molecular orbital (HOMO) mainly distributes on the dithiin linkage and benzene rings, while the benzoquinone groups dominate the lowest unoccupied molecular orbital (LUMO). This result is consistent with the energy band calculation.

The electrostatic potential (ESP) of CityU‐9 was then calculated to determine the possible Na^+^ insertion positions.^[^ ^37^, ^38^ ^]^ As shown in Figure 3c, the minima of the ESP mainly distributes around the oxygen atoms. Due to the spatial closure of the oxygen atoms, the first‐inserted Na atom interacts with two adjacent oxygen atoms and two adjacent sulfur atoms (Figure 3d). The binding energy of this intraction is −3.442 eV, which demonstrates the interaction capability of Na ions.^[^ ^39^ ^]^ Also, the differential charge density (DCD) indicates that the charge density of the oxygen atoms and sulfur atoms increases while the charge density of sodium atoms decreases after the insertion. Another type of interaction occurs between Na ion and three atoms including one oxygen atom and two sulfur atoms. The binding energy is −2.864 eV, which is slightly smaller than the former interaction mode (Figure S4, Supporting Information). After all the carbonyl moieties are bound, further insertions of the Na ions share the occupied oxygen atoms and sulfur atoms (Figure 3e; Figures S5,S6, Supporting Information). Nevertheless, the ESP distribution of CityU‐9‐11Na shows no negative charge within the COP skeleton so that it is impossible for the addition of 12^th^ Na ion (Figure S7, Supporting Information). Therefore, the theoretical capacity of CityU‐9 is calculated to be 349 mAh g^−1^, corresponding to insertion of 11 Na ions. The Bader charge analysis demonstrates that the insertion of Na ions apparently increases the charge of the interacted sulfur atoms, which further proves the participance of sulfur in the redox reaction (Figure S8, Supporting Information).

### 2.3 Electrochemical and Battery Performance

**Figure 4:**
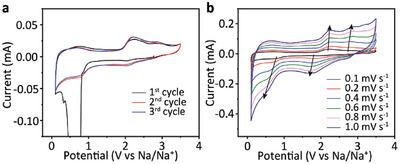
a) Cyclic voltammetry (CV) curves of first three cycles of CityU‐9 at 0.1 mV s−1. b) CV curves of CityU‐9 measured at different scan rates.

The electrochemical performance of CityU‐9 was then investigated. According to the cyclic voltametric (CV) curves scanned at 0.1 mV s^−1^, the broad peak located at 0.1–1.0 V during the first anodic process is attributed to the formation of solid electrolyte interphase (SEI) on the electrode surface (**Figure** **4**a). Three couples of overlapped redox peaks located at ≈0.7, 2.1, and 2.8 V demonstrate the reversible redox reaction of CityU‐9 electrode. From the CV curves obtained at different scan rates ranging from 0.1 to 1.0 mV s^−1^ (Figure 4b), we are able to reveal the diffusion kinetics of Na ions based on the following equations:^[^ ^40^, ^41^ ^]^

(1) $$i=a\upsilon^{b}$$

(2) logi=blogυ+loga

where *i* and *ν* are the peak current of the redox peak and the sweep rate, respectively. When b = 0.5, the process is diffusion controlled; whereas the process is pseudocapacitive when b = 1. By linear fitting the log*i* and log*ν* of the redox peaks, the slopes of the fitting curves are distributed from 0.71 to 0.96, demonstrating a supercapacitor‐like behavior of CityU‐9 (Figure S9, Supporting Information). This result indicates fast kinetics for Na^+^ diffusion. The contribution of pseudocapacitance was further evaluated based on the equation 3:^[^ ^40^, ^41^ ^]^

(3) $$\frac{i}{\upsilon^{0.5}}=k_{1}\;\upsilon^{0.5}+k_{2}$$

After acquiring the *k*_1_ values at the different potential by linear fitting *i*/*ν* ^0.5^ and *ν* ^0.5^, the *k*_1_ *ν* is the simulated current at a certain sweep rate. The contribution of the pseudocapacitance can be estimated by area ratio of the simulated curve to the CV curve. While the scan rate increases from 0.1 to 1.0 mV s^−1^, the contribution of pseudocapacitance increases from 60.7% to 83.5%, further confirms the supercapacitor‐like Na^+^ storage kinetics, which is beneficial to good rate performance (Figure S10, Supporting Information).

**Figure 5:**
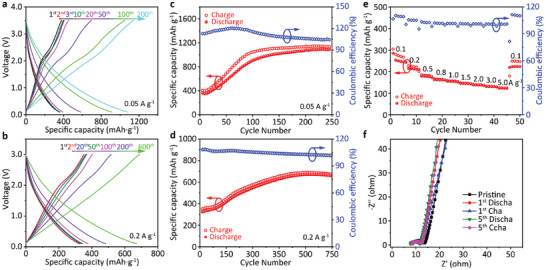
The charging and discharging curves of the CityU‐9‐based half‐cell SIBs at particular cycles using the current density of a) 0.05 A g^−1^ and b) 0.2 A g^−1^. Cycling stability of CityU‐9‐based half‐cell SIBs at the current density of c) 0.05 A g^−1^ and d) 0.2 A g^−1^. e) Galvanostatic charge/discharge measurements of CityU‐9‐based half‐cell SIB at 0.1, 0.2, 0.5, 0.8, 1.0, 1.5, 2.0, 3.0, and 5.0 A g^−1^. f) Nyquist plots of the pristine CityU‐9‐based half‐cell SIB and after 1st and 5th charging/discharging processes.

On the basis of electrochemical property of CityU‐9, we attempted to assemble half‐cell SIB utilizing CityU‐9 as the active electrode material accompanying with Na metal electrode. It delivers a high initial charge / discharge specific capacity of 378 / 355 mAh g^−1^ in the voltage range of 0.1–3.5 V and at current density of 50 mA g^−1^ (**Figure** **5**a). After removing the contribution of conductive carbon, the individual contribution of CityU‐9 is 294/271 mAh g^−1^ for charge/discharge specific capacity. Interestingly, after few cycles of slight decrease, the discharge specific capacity rapidly increases to 1009 mAh g^−1^ and then becomes stable (Figure 5c, the contribution of conductive carbon has been eliminated). Similarly, the discharge capacity of half‐cell SIB operated at a high current density of 200 mA g^−1^ increases from 241 mAh g^−1^ for the initial cycle to 588 mAh g^−1^ for the 600^th^ cycle even in a narrowed voltage range of 0.1–3.0 V (Figure 5b). This half‐cells also exhibits excellent cycling stability with nearly undiminished capacity after 650 cycles. (Figure 5d). Even at ultrahigh current density of 3 A g^−1^, no capacity loss is observed before 1000 cycles (Figure S11, Supporting Information). Unlike the common organic electrodes,^[^ ^17^, ^28^ ^]^ no obvious drops of the specific capacity are observed in the first several cycles of these half‐cell SIBs, illustrating the success of our robust skeleton design strategy. Besides, Figure 5e shows superior rate capability at a wide current density range of 0.1 to 5 A g^−1^ between 0.1 and 3.5 V. Specific capacities of 253, 179, and 162 mAh g^−1^ are obtained at the current density of 0.1, 0.5, and 1 A g^−1^, respectively. Even at very high current density of 5 A g^−1^, which is >13 C, 49% of the initial specific capacity can be retained (124 mAh g^−1^). Such excellent rate capability is in accordance with the pseudocapacitance contribution. Furthermore, the electrochemical impedance spectroscopies (EIS) of the Na//CityU‐9 cell at different charge and discharge states with a wide frequency range from 100 mHz to 100 kHz were tested. The Nyquist plots illustrate that the charge transfer resistance (*R*_ct_, high‐frequency regions) of CityU‐9 electrode is low to 4.3 Ω, and becomes even lower after several cycles (Figure 5f). Such low resistance should be resulted from the assistance of dithiin linkages with conduction improving capability. The straight lines in the low‐frequency region show inclination angles much larger than 45°, indicating pseudocapacitance‐controlled process, which enables fast reaction kinetics on the electrode surface and excellent rate performance of CityU‐9‐based electrodes.

**Figure 6:**
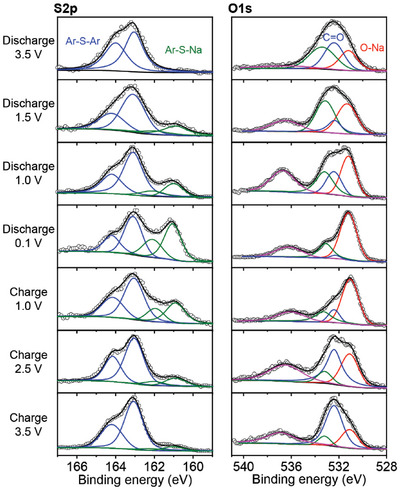
S2p (left) and O1s (right) ex situ XPS spectra of CityU‐9 electrode at different voltage during the discharging and charging processes.

To uncover the mechanism of the Na ion storage and the abnormal high specific capacity, ex situ X‐ray photoelectron spectroscopy (XPS) curves of the electrode at different charging and discharging states were measured. As shown in **Figure** **6**, a couple of new peaks of S2p appears at 161.0 (2p 3/2) and 162.1 eV (2p 1/2) during the discharging process, demonstrating the formation of S─Na bonds.^[^ ^42^ ^]^ This bonding is reversible as the signal gradually disappears during the charging process. Correspondingly, a new peak appears at 531.1 eV in the O1s spectra during the discharging process, referring to the O─Na bonding.^[^ ^43^ ^]^ Besides, the peak at ≈536 eV refers to the Na KLL (Augar) signal.^[^ ^44^ ^]^ Thus, the XPS results clearly illustrate the synergistic interactions of dithiin and benzoquinone units to Na^+^ during the charging/discharging processes and agree with the theoretical calculation. To reveal the capacity increase during the cycling, the half‐cell after cycling was disassembled and the XPS of the CityU‐9 electrode was tested afterward. To our surprise, a new couple of peaks appear at ≈169.0 eV in the S2p curve, which could be attributed to the sulfone species (Figure S12, Supporting Information).^[^ ^35^ ^]^ The sulfone group has two S=O double bonds, which may exhibit similar multiple‐electron redox behavior to nitro groups.^[^ ^45^, ^46^ ^]^ The relatively high coulombic efficiency (CE, ratio of charging capacity to discharging capacity) in the capacity increase period should be ascribed to the oxidation of the thioether groups, which apparently decreases to ≈100% after reaching the maximum of the capacity. Apart from the former reason, the gradual exposure of active sites to the electrolyte,^[^ ^19^ ^]^ as well as sodiation of carbon backbone at low voltage^[^ ^47^ ^]^ are also responsible for the capacity increase. The fully sodiated COP can provide an ultrahigh theoretical capacity of 1306 mAh g^−1^.

Since CityU‐9 is redox‐active both at high potential and low potential (vs Na/Na^+^), the cathodic and anodic performance of the CityU‐9 electrode in SIBs was then tested separately. When CityU‐9 electrode was used as the cathode, an initial discharging specific capacity of 120 mAh g^−1^ is achieved at the current density of 50 mA g^−1^ (Figure S13, Supporting Information). Similar to the former half cells, the specific capacity of the CityU‐9 cathode‐based cell slightly decreases during the initial several cycles and gradually increases to 318 mAh g^−1^ at the 625^th^ cycle. This phenomenon should also be attributed to the oxidation of dithiin caused Na^+^ coordinating position increment and gradual activation of the COP. For the anodic performance of CityU‐9 electrode, the discharge‐first process is necessary to load Na ions on the CityU‐9 skeleton. The maximum specific capacity of the CityU‐9 anode can reach 297 mAh g^−1^ at current density of 20 mA g^−1^ in the voltage range of 0.05 to 2.0 V, which has eliminated the contribution of conductive carbon (Figure S14, Supporting Information). When the current density increases to 50 mA g^−1^, the initial capacity decreases to 215 mAh g^−1^ but with improved CE.

**Figure 7:**
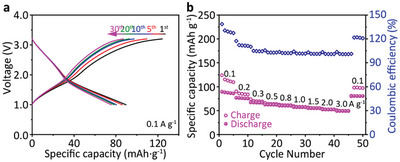
a) The charging and discharging curves of the CityU‐9‐based symmetric full‐cell SIB at particular cycles using current density of 0.1 A g^−1^. b) Galvanostatic charge/discharge measurement of CityU‐9‐based symmetric full‐cell SIB at 0.1, 0.2, 0.3, 0.5, 0.8, 1.0, 1.5, 2.0, and 3.0 A g^−1^.

Encouraged by the large operation potential range of CityU‐9 redox sites and good performance of its half‐cell SIBs, we tried to fabricate symmetric full‐cell SIB based on CityU‐9 cathode and presodiated CityU‐9 anode. Notably, this is the first example of all‐COP full‐cell SIB to date. High reversible specific capacity of 90 mAh g^−1^ is achieved in the initial cycle and it reduces slowly in the first 30 cycles (**Figure** **7**a). Besides, the excellent rate capability can be observed in Figure 7b, where a high specific capacity of 49 mAh g^−1^ can be retained at the high current density of 3 A g^−1^, corresponding to 55% of the initial capacity at 0.1 A g^−1^. At this high current density, a full charging process only requires 36 s. Furthermore, 45% of capacity retention can be obtained after 1000 cycles of charging and discharging at current density of 0.1 A g^−1^ (Figure S15, Supporting Information). Such excellent cycling stability and rate performance is resulted from the intrinsic insolubility of CityU‐9 and high pseudocapacitive contribution at high current density.

## 3 Conclusion

In this research, a novel COP (CityU‐9) containing benzoquinone moieties and dithiin linkages is designed and synthesized. With the integrated advantages of the insoluble robust skeleton, abundant redox‐active sites, and improved conductivity, ultrahigh specific capacity up to 1009 mAh g^−1^ as well as excellent rate capability and cycling stability are achieved solely by CityU‐9 in the half‐cell battery. The outstanding battery performance is proven to be generated from the co‐interaction of sulfur and oxygen atoms with sodium ions, as well as the gradual oxidation of sulfur atoms that provides more interaction sites than pristine dithiin linkages. Due to the wide potential window of CityU‐9, good cathodic and anodic SIB performance are also realized. Furthermore, the all‐COP symmetric full‐cell SIB is fabricated for the first time based on the CityU‐9 cathode and presodiated CityU‐9 anode. An initial specific capacity of 90 mAh g^−1^ and 45% capacity retention after 1000 cycles were achieved. Moreover, remarkable rate performance of 55% capacity retaintion at high current density of 3 A g^−1^ is also obtained. Such good SIB performance illustrates the great potential of sulfur‐containing COPs in the ultrahigh capacity metal‐ion batteries. This work also provides a novel design strategy of high performance electrode materials for energy storage applications.

## Conflict of Interest

The authors declare no conflict of interest.

## Supporting information

Supporting Information

## Data Availability

The data that support the findings of this study are available in the supplementary material of this article.
