# Supplementary material for: A Dithiin‐Linked Covalent Organic Polymer for Ultrahigh Capacity Half‐Cell and Symmetric Full‐Cell Sodium‐Ion Batteries
Source: Adv Sci (Weinh). 2023 Sep 25;10(32):2304497. doi: 10.1002/advs.202304497 (PMC10646242; doi:10.1002/advs.202304497)
Supplement: Supplementary file 1 — Supporting Information [file ADVS-10-2304497-s001.pdf]

ADVANCED SCIENCE
Open Access

Supporting Information

for *Adv. Sci.*, DOI 10.1002/advs.202304497

A Dithiin-Linked Covalent Organic Polymer for Ultrahigh Capacity Half-Cell and Symmetric Full-Cell Sodium-Ion Batteries

Shen Xu, Chenchen Wang, Tianyi Song, Huiying Yao, Jie Yang, Xin Wang, Jia Zhu, Chun-Sing Lee* and Qichun Zhang**

1. Supplementary Methods

Materials and solvents

Unless other mentioned, all the materials and solvents used in the experiments were purchased from TCI chemical, Aladdin Biochemical, Zhengzhou Alfa, J&K Chemical, and Anaqua without further purification.

Measurements

Fourier Transform infrared (FT-IR) spectra were recorded on a on a PerkinElmer Spectrum Two FTIR Spectrometer. Solid-state ^{13}C cross polarization-total sideband suppression nuclear magnetic resonance (CP-TOSS NMR) spectrum was tested on a Bruker Avance NEO 400WB. XPS patterns were measured using a Thermo Fisher ESCALAB Xi^+ X-ray Photoelectron Spectrometer (Al Kα: λ = 0.834 nm) under ambient conditions. The thermogravimetric curves were measured on a PerkinElmer STA 6000 using ceramic pan as the container with a heating rate of 10°C·min^{-1} and a nitrogen flow rate of 20 cm^3·min^{-1}. Diffuse reflectance spectrum (DRS) was recorded on a Hitachi UH4150 UV-VIS-NIR Spectrophotometer using an integrating sphere. Cyclic voltammetry (CV) tests were carried out on an IviumStat electrochemical workstation with a voltage range of 0.1-3.5 V and a scan rate of 0.1 mV s^{-1}. The galvanostatic charge/discharge measurements were performed on a NEWARE test system (NEWARE, CT4008) at current densities of 20, 100, 200, 400, 500, 800, 1000, 1500, 2000, 3000 and 5000 mA g^{-1}. Electrochemical impedance spectroscopy (EIS) was obtained by applying a sine wave with an amplitude of 10 mV in the frequency range from 100 kHz to 100 mHz on an IviumStat electrochemical workstation.

Theoretical calculation

All calculations were performed using the Vienna *ab* initio simulation package (VASP).[1] The generalized gradient approximation with the Perdew–Burke–Ernzerhof exchange-correlation functional was adopted.[2] The semiempirical DFT-D3 correction *via* Grimme's scheme was considered to describe van der Waals interactions.[3] The projector augmented wave potentials were used with a kinetic energy cutoff of 500 eV. All geometries were relaxed until the total energy difference less than 10^{-5} eV and the force on each atom less than 0.01 eV Å^{-1}. The Brillouin zone were sampled by using a gamma centered 5×1×1 mesh for structural

optimization and 8×2×2 mesh for density of states calculation. The HSE06 functional[4] was adopted to calculate the band structure of the unit cell of COP. The molecular electrostatic potential (MESP) method was used to determine the sodiation sites of COP in the Multiwfn 3.6 software.[5]

Preparation

Synthesis of benzo[5,6][1,4]dithiino[2,3-b]thianthrene-6,13-dione (model compound, BTBQ).[6,7]

Benzene-1,2-dithiol (BDT, 100 mg, 0.7 mmol), 2,3,5,6-tetrafluoro-1,4-benzoquinone (TFBQ, 54 mg, 0.3 mmol), and potassium carbonate (249 mg, 1.8 mmol) were charged in a flask. After evacuating and filling with argon, N,N'-dimethylformamide (5 mL) was added and stirred at 90°C for 24 h. The product was purified by column chromatography to give a dark red solid (19.3 mg, 16.8%).

Preparation of CityU-9.

A Schleck tube was charged with 1,2,3,4,5,6-benzenehexathiol (BHT, 40.0 mg), TFBQ (40.0 mg), sodium carbonate (122.3 mg), and n-butanol (2 mL). The mixture was frozen to 78 K in a liquid nitrogen bath and evacuated for 10 min, followed by warming to room temperature after sealing. After another two freeze–pump–thaw cycles, the mixture was heated to 120°C and reacted for 72 h. The crude produce was filtered and washed with water, dimethylformamide, tetrahydrofuran, and dichloromethane to yield a brown solid (49.4 mg, 79.5%).

Preparation of CityU-9-based cathode.

CityU-9, Ketjen Black, and polyvinylidene fluoride (PVDF) were mixed in N-methyl-2-pyrrolidone (NMP) with a mass ratio of 5:4:1. The obtained slurry was pasted onto an Al foil and dried at 80°C for 12 h in a vacuum oven. The loaded mass of the active material is about 0.12 mg cm^{-2}.

Fabrication of a half SIB coin cell with the CityU-9-based cathode.

A sodium disk was first pressed to the stainless steel spacer. Then an O-ring was placed on the smaller case and pressed against the case. Next, a spring, the assembled stainless steel spacer and sodium disk were placed on the O-ring sequentially, with the sodium disk facing upwards. After that, a separator (glass fiber) was placed on top of the sodium disk as centered as possible. 200 μL of 1M sodium hexafluorophosphate ($NaPF_6$) in diglyme as the electrolyte was then

dropped onto the separator. Next, the former obtained CityU-9-based cathode was placed on top, with the cast film facing the sodium disk and centered as much as possible with the sodium disk to avoid uneven current densities. Finally the larger case was placed on top and the coin cell was packed with 0.8 kPa pressure.

Fabrication of CityU-9-based anode and presodiated CityU-9-based anode.

CityU-9, Ketjen Black, and polyvinylidene fluoride (PVDF) were mixed in N-methyl-2-pyrrolidone (NMP) with a mass ratio of 5:4:1. The obtained slurry was pasted onto an Al foil and dried at 80°C for 12 h in a vacuum oven, and thereby the (pristine) CityU-9-based anode is obtained. The obtained (pristine) CityU-9-based anode was combined with a Na electrode to fabricate a half cell battery. After performing a discharging process at 50 mA g^{-1} for about 10 hours, the cell was disassembled in the glovebox to isolate the presodiated CityU-9-based electrode as the anode of the full cell.

Fabrication of the half SIB coin cell with the CityU-9-based anode.

An O-ring was placed on a smaller cap and pressed against the case. Next, a spring, a stainless steel spacer and the (pristine) CityU-9-based anode were placed on the o-ring sequentially, with the cast film of the anode facing upwards. After that, a separator (glass fiber) was placed on top of the anode as centered as possible. 200 μL of 1M sodium hexafluorophosphate ($NaPF_6$) in diglyme as the electrolyte was then dropped onto the separator. Next, a sodium disc cathode was placed on top, with the cast film facing the anode and centered as much as possible with the anode to avoid uneven current densities. Finally the larger case was placed on top and the coin cell was packed with 0.8 kPa pressure.

Fabrication of the full SIB coin cell with the CityU-9-based cathode and the presodiated CityU-9-based anode.

An O-ring was placed on a smaller cap and pressed against the case. Next, a spring, a stainless steel spacer and the presodiated CityU-9-based anode were placed on the O-ring sequentially, with the cast film of the anode facing upwards. After that, a separator (glass fiber) was placed on top of the anode as centered as possible. 200 μL of 1M sodium hexafluorophosphate ($NaPF_6$) in diglyme as the electrolyte was then dropped onto the separator. For pre-sodiation of anode, we discharged the BHT-BQ anode to a discharge capacity of 158 mAh g^{-1} for the 2nd cycle in a voltage window of 0.01-2.0 V. There should be 5.0 mol Na^+ in CityU-9 anode after pre-sodiation process, which can ensure that the full cell has sufficient sodium source. Next, the

CityU-9-based cathode was placed on top, with the cast film facing the anode and centered as much as possible with the anode to avoid uneven current densities. Finally the larger case was placed on top and the coin cell was packed with 0.8 kPa pressure.

Supplementary Figures

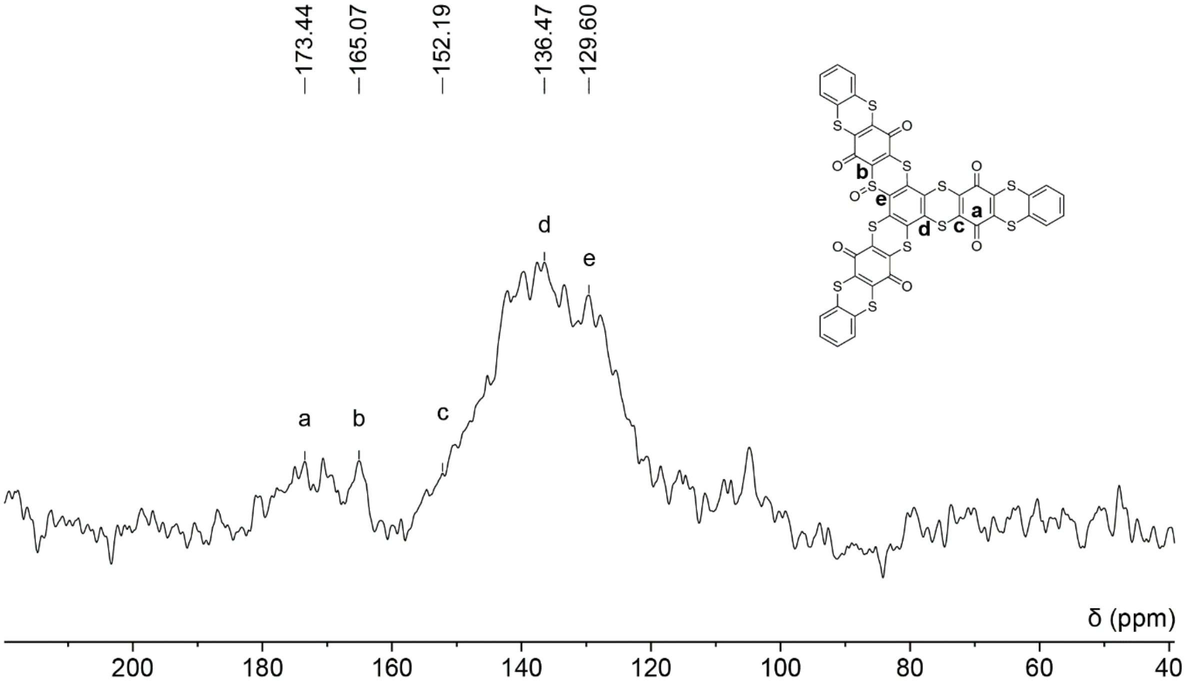

Figure S1. (a) Solid-state ^{13}C-NMR spectrum of CityU-9.

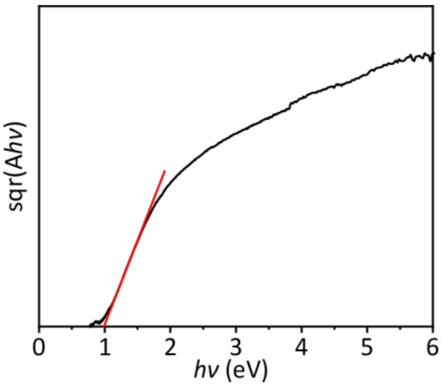

Figure S2. (a) Tauc plot of CityU-9.

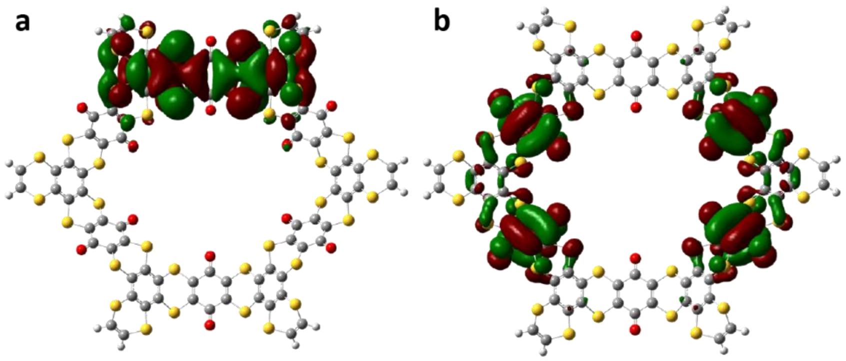

Figure S3. (a) Highest occupied molecular orbital (HOMO) and (b) lowest unoccupied molecular orbital (LUMO) distribution of CityU-9.

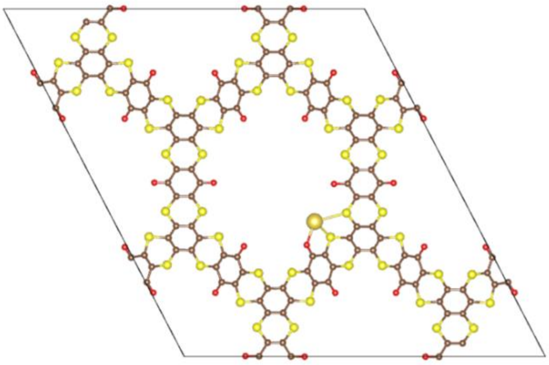

Figure S4. Interaction mode of Na ion with one oxygen atom and two sulfur atoms.

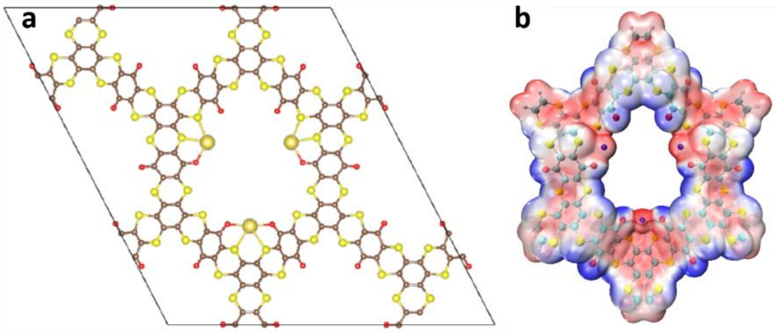

Figure S5. The structure and ESP distribution of CityU-9 after adding 3 Na ions.

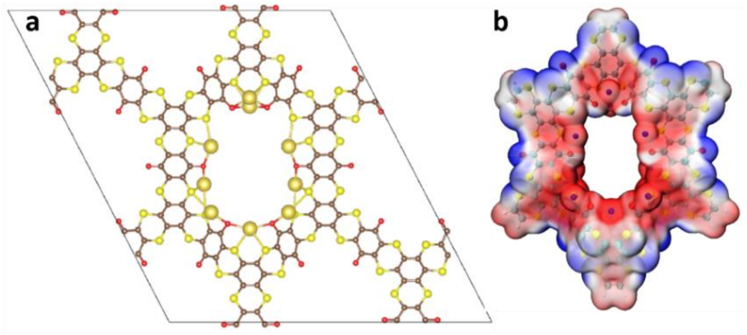

Figure S6. The structure and ESP distribution of CityU-9 after adding 9 Na ions.

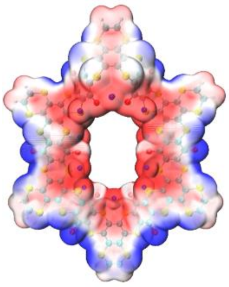

Figure S7. The ESP distribution of CityU-9 after adding 11 Na ions.

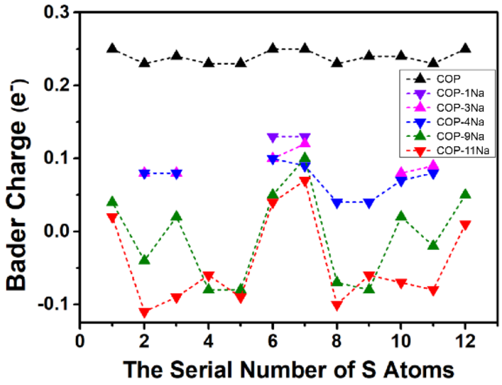

Figure S8. Bader charge analysis of CityU-9 after adding different numbers of Na ions.

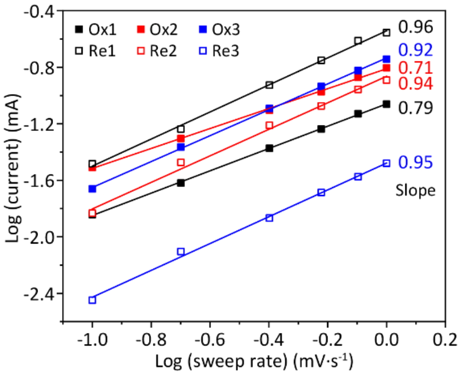

Figure S9. Corresponding linear fit of the log (peak current) vs log (sweep rate) at 0.1, 0.2, 0.4, 0.6, 0.8, and 1.0 mV s^{-1}.

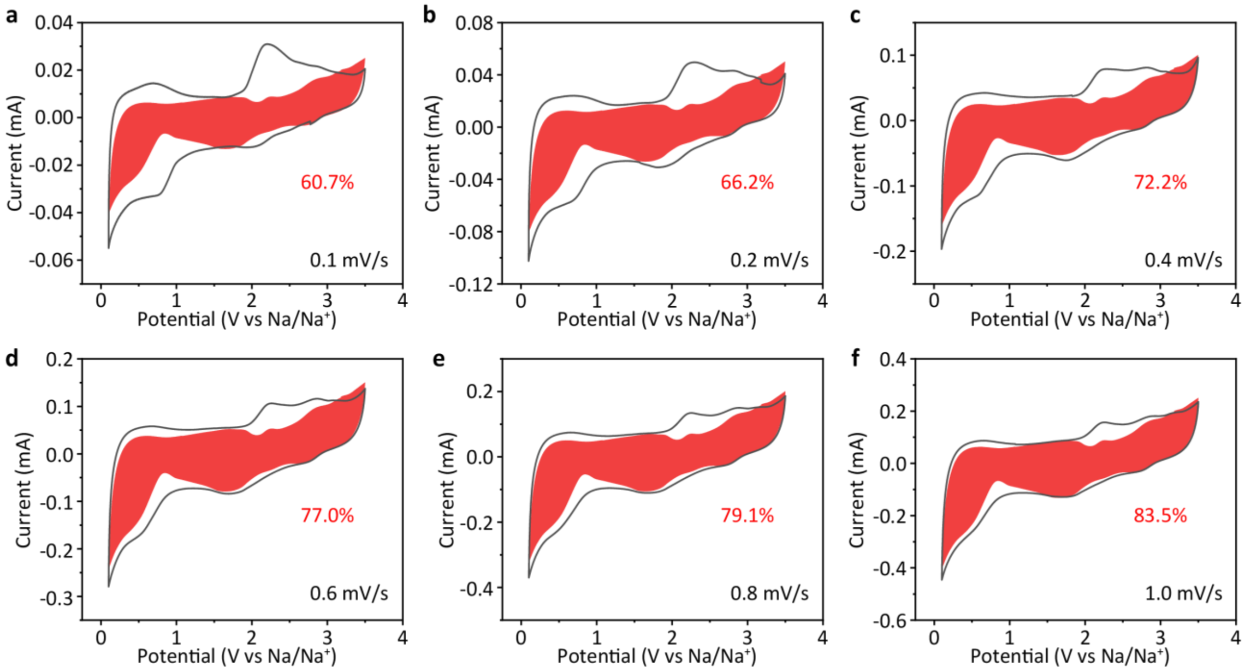

Figure S10. Pseudocapacitance contribution at sweep rate of (a) 0.1 mV·s^{-1}, (b) 0.2 mV·s^{-1}, (c) 0.4 mV·s^{-1}, (d) 0.6 mV·s^{-1}, (e) 0.8 mV·s^{-1}, and (f) 1.0 mV·s^{-1}.

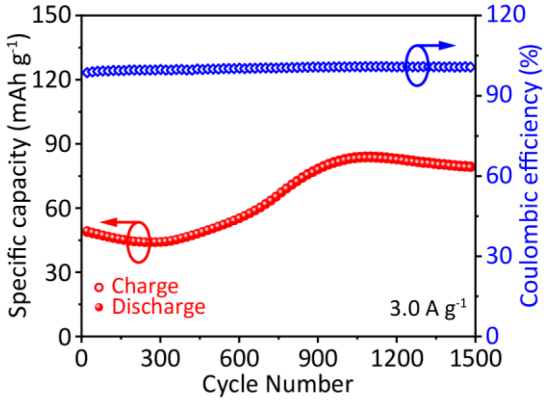

Figure S11. Cycling stability of CityU-9-based half-cell SIBs at current density of 3.0 A·g^{-1}.

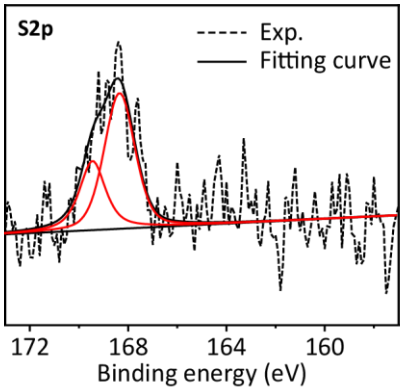

Figure S12. S2p XPS spectra of CityU-9 electrode after hundreds of discharge/charge cycles.

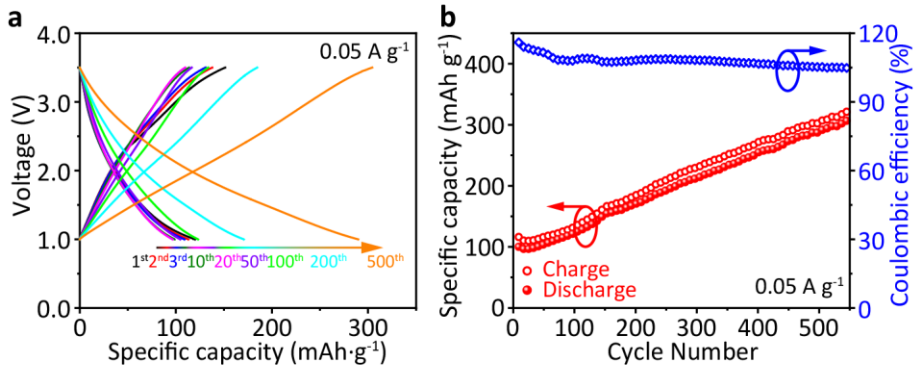

Figure S13. (a) Charging and discharging curves of CityU-9 cathode-based half-cell SIB of specific cycles. (b) Cycling stability of CityU-9 cathode-based half-cell SIB at current density of 0.05 A·g^{-1}.

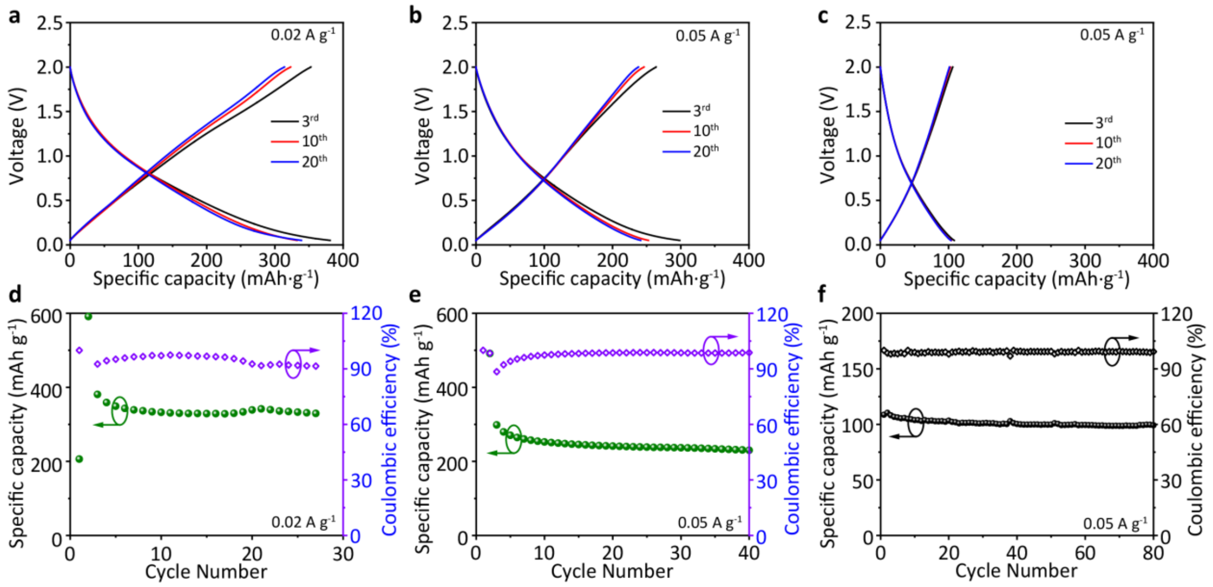

Figure S14. (a) Charging and discharging curves of CityU-9 anode-based half-cell SIB at (a) 0.02 A·g^{-1} and (b) 0.05 A·g^{-1}, and (c) Ketjen Black (KB) anode-based half-cell SIB at 0.05 A·g^{-1} in the 3rd, 10th, and 20th cycles. Cycling stability of CityU-9 anode-based half-cell SIB at current density of (d) 50 mA·g^{-1}, (e) 50 mA·g^{-1}, and (f) KB anode-based half-cell SIB at 0.05 A·g^{-1}.

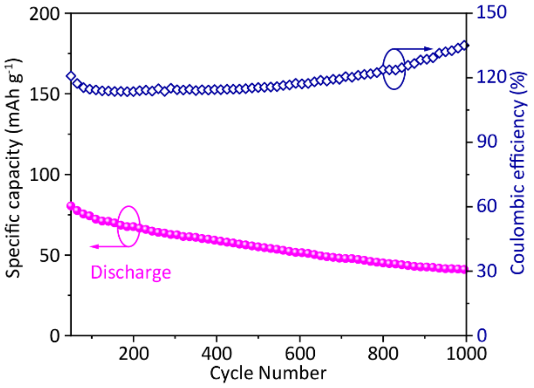

Figure S15. Cycling stability of symmetric full-cell SIB based on CityU-9 cathode and presodiated anode at current density of 0.1 A·g^{-1}.

References

[1] G. Kresse, J. Furthmüller, *Phys. Rev. B* **1996,** *54*, 11169.

[2] J. P. Perdew, K. Burke, M. Ernzerhof, *Phys. Rev. Lett.* **1996,** *77*, 3865.

[3] S. Grimme, *J. Comput. Chem.* **2006,** *27*, 1787.

[4] J. Heyd, G. E. Scuseria, M. Ernzerhof, *J Chem. Phys.* **2003,** *118*, 8207.

[5] L. Liu, L. Miao, L. Li, F. Li, Y. Lu, Shang, J. Chen, *J Phys. Chem. Lett.* **2018,** *9*, 3573.

[6] [1] K. Takagi, A. Mizuno, A. Iwamoto, M. Furusyo, M. Matsuoka, *Dyes Pigm.* **1998**, *36*, 35.

[7] S. Matsumoto, H. Miura, J. Mizuguchi, *Dyes Pigm.* **2002**, *52*, 9.
